# Homœopathic Bibliography of the United States

**Published:** 1892-09

**Authors:** 


Homœopathic Bibliography of the United States, from the year 1825 to the year 1891, inclusive. Carefully compiled and arranged by Thomas Lindsley Bradford, M. D. Philadelphia: Bœricke & Tafel, 1011 Arch Street, 1892. Price, cloth, $3.50; half morocco, $4.50, net.

"This book has been compiled because it has seemed fitting that a permanent record should be made of the homœopathic books and institutions, past and present, of the United States.

"With every year it is becoming more difficult to obtain reliable facts concerning the early history of Homœopathy in America."

The foregoing lines are quoted from the opening paragraph of the preface of this book, and concisely state the motive of the author.

Our readers will remember an announcement of the forthcoming issue of this work months ago. The book in nowise falls below the promises of the announcement. It is a most valuable addition to homœopathic literature because it is a history of homœopathic literature and institutions.

It is divided into two parts: Part I, List of homœopathic books and pamphlets in alphabetical order, list of books against Homœopathy, magazines, directories, homœopathic publishers, libraries, and previous American Bibliography. Part II, Condensed histories, data, and bibliography of homœopathic societies, colleges, hospitals, asylums, homes, sanitariums, asylums for the insane, dispensaries, pharmacies, life insurance, legislation, etc. Thus the reader will gain an idea of how very comprehensive is its scope.

Dr. Bradford certainly deserves the thanks of every homœopathic physician for such a labor of love. May he have a substantial token of the appreciation of the profession by an early exhaustion of the edition.

